# Diagnostic Challenges in a Case of Suspected Breast Cancer with Low FDG Uptake and an Incidental Thyroid Lesion: A Case Report and Literature Review

**DOI:** 10.1055/s-0045-1809310

**Published:** 2025-05-19

**Authors:** Raydel Briankwee Amalo, Yustia Tuti, Ayu Rosemeilia Dewi

**Affiliations:** 1 Department of Nuclear Medicine and Theranostic Molecular, School of Medicine, Universitas Padjajaran, Hasan Sadikin General Hospital, Bandung, Indonesia; 2 Department of Nuclear Medicine and Theranostic Molecular, Dharmais Cancer General Hospital, Jakarta, Indonesia

**Keywords:** bidirectional breast and thyroid cancer, breast cancer, dual malignancy, incidentaloma thyroid, low FDG Uptake

## Abstract

**Introduction:**

The coexistence of multiple malignancies presents diagnostic and therapeutic challenges. Breast and thyroid cancers are among the most frequently diagnosed malignancies in women, and studies suggest a potential bidirectional association. While fluorodeoxyglucose (FDG)-positron emission tomography (PET)/computed tomography (CT) is a valuable imaging modality for evaluating breast cancer, its sensitivity in detecting low-metabolic subtypes remains limited. Additionally, incidental FDG-avid thyroid lesions require further evaluation due to their potential malignancy risk.

**Case Report:**

We present a 61-year-old female with a suspected left breast malignancy, suggestive of luminal A subtype, showing low FDG uptake (maximum standardized uptake value [SUVmax] 2.0) on PET/CT, despite mammographic and ultrasound findings suggestive of malignancy (Breast Imaging-Reporting and Data System 4A and V). Additionally, an incidental left thyroid lesion (4.0 × 3.8 cm, SUVmax 3.4) with calcifications was detected, raising suspicion for malignancy. The discordant imaging findings in this case highlight the limitations of FDG-PET/CT and emphasize the necessity of multimodal imaging and histopathological confirmation.

**Conclusion:**

This case underscores the importance of integrating multiple imaging modalities for accurate diagnosis. While PET/CT is useful for systemic staging, its limitations in detecting certain breast cancer subtypes necessitate complementary imaging techniques and histopathological confirmation. The incidental thyroid lesion also required further assessment, reinforcing the need for a comprehensive diagnostic approach.

## Introduction

Breast and thyroid cancers are commonly diagnosed malignancies in women, with epidemiological evidence suggesting a bidirectional association. 1 Fluorodeoxyglucose (FDG)-positron emission tomography (PET)/computed tomography (CT) is widely used in breast cancer staging, particularly for aggressive subtypes, but has limitations in detecting low-metabolic tumors. Incidental FDG-avid thyroid lesions require careful assessment due to their malignancy potential. 2 This case underscores the need for a multidisciplinary approach in diagnosing coexisting malignancies.

## Case Presentation

A 61-year-old female presented with a palpable left breast lump persisting for 6 months. She had a history of hypertension managed with amlodipine. Physical examination revealed no significant lymphadenopathy in the bilateral axillae and cervical regions.

### Imaging Findings

Breast ultrasound (December 2024): Left breast lesion (1.15 × 1.25 × 1.43 cm), irregular margins, internal vascularization, and Breast Imaging-Reporting and Data System (BIRADS) V classification.

Mammography (February 2025): Indeterminate left breast nodule (0.6 × 0.6 cm) with amorphous microcalcifications, BIRADS 4A classification.

**Fig. 1:**
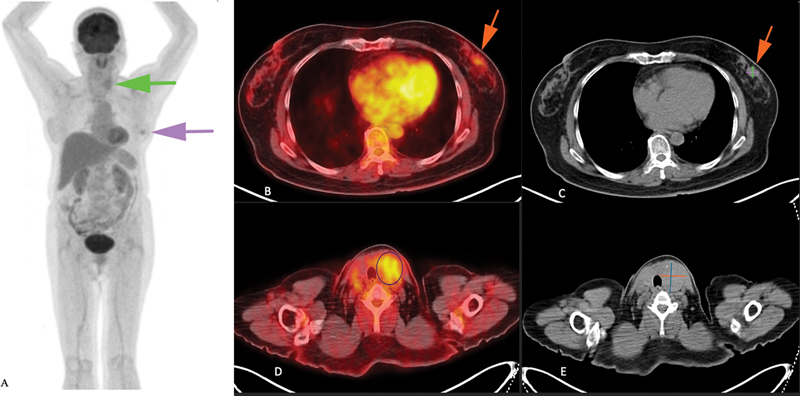
( **A** ) Maximum intensity projection (MIP) positron emission tomography (PET)/computed tomography (CT) image showing increased fluorodeoxyglucose (FDG) uptake in the left breast and left thyroid lobe. ( **B** ) Axial fused PET/CT image demonstrating FDG-avid lesion in the left breast. ( **C** ) Corresponding axial CT image revealing a left breast lesion measuring 1.7 × 1.5 cm. ( **D** ) Axial fused PET/CT image demonstrating FDG uptake in the left thyroid lobe. ( **E** ) Corresponding axial CT image showing a left thyroid nodule measuring 4.0 × 3.8 cm.

PET/CT (February 2025): Left breast lesion (1.7 × 1.5 cm) with low FDG uptake (maximum standardized uptake value [SUVmax] 2.0), metabolically inactive axillary lymphadenopathy, and an incidental left thyroid lesion (4.0 × 3.8 cm, SUVmax 3.4) ( Fig. 1 ).

Subsequent thyroid ultrasonography (US) revealed a nodule classified as Thyroid Imaging Reporting and Data System 5, indicating a high suspicion of malignancy.

## Discussion

Mammographic (BIRADS 4A) and ultrasonographic (BIRADS V) findings suggested malignancy, but PET/CT demonstrated low FDG uptake (SUVmax 2.0), inconsistent with aggressive breast carcinoma. Despite a low SUVmax, biopsy remains crucial, as mammographic features such as microcalcifications and irregular edges strongly correlate with malignancy. PET/CT has limitations in detecting microcalcifications and low-FDG tumors, making mammography more reliable for early malignancy detection. Consequently, histopathologic biopsy remains the gold standard.

According to the National Comprehensive Cancer Network 2024 guidelines, BIRADS 4A lesions require biopsy regardless of SUVmax due to a strong malignancy correlation. 3 The European Association of Nuclear Medicine-Society of Nuclear Medicine and Molecular Imaging 2024 guidelines highlight that FDG-PET/CT is highly effective for triple-negative breast cancer (TNBC) and HER2+ cancers but less so for estrogen receptor-positive (ER + ) tumors (luminal A/B) due to lower glucose metabolism. 2 Invasive lobular carcinoma also exhibits lower FDG uptake than no special type subtypes, reducing PET sensitivity. 3 A meta-analysis found that high SUVmax is associated with poorer outcomes (hazard ratio 2.65; 95% confidence interval [CI] 1.31–5.37; *p* = 0.007), though thresholds vary (5.5–11.1 for primary tumors, 2.2–2.8 for axillary nodes). 4 In this case, an SUVmax of 2.0 suggests lower aggressiveness.

FDG-PET/CT plays a key role in staging stage IIB to III breast cancer, with superior accuracy in axillary node and distant metastasis detection. It is crucial for assessing neoadjuvant therapy response, particularly in TNBC and HER2+ cases, and is valuable for detecting recurrence before curative interventions. 3 5 Studies report that FDG-avid breast lesions with calcifications have a malignancy rate of 67.8%, increasing to 76.2% if categorized as BIRADS ≥ 4 on CT. 6 Moreover, FDG uptake without calcification but with irregular margins is also strongly associated with malignancy. Dong et al found that 88.2% of malignant lesions with FDG uptake had irregular margins on CT. 7

SUVmax does not always indicate benignity. Luminal A (3.32 ± 2.60) and luminal B (HER2-negative) (4.74 ± 2.69) tumors exhibit lower SUVmax than TNBC (9.86 ± 3.24) due to metabolic differences. Luminal tumors rely on oxidative phosphorylation, whereas TNBC and HER2+ tumors depend on anaerobic glycolysis. A cutoff SUVmax of ≤ 5.46 predicts luminal tumors but lacks specificity, whereas SUVmax of ≥ 6.33 is more reliable for TNBC classification. Despite the low SUVmax of 2.0 in this case, mammography findings (BIRADS 4A) still indicate possible malignancy. 8

In thyroid cancer, incidental FDG uptake suggests a higher malignancy risk. 9 US and physical examination are crucial for evaluation, with irregular margins, microcalcifications, and infiltrative borders being strong malignancy indicators. Large (> 4 cm) or solitary nodules carry a malignancy risk of up to 19.3%. 10 Hard, fixed nodules or cervical lymphadenopathy further increase suspicion, while vocal cord immobility suggests recurrent laryngeal nerve involvement (positive predictive value 100%). 11 In this case, the left lobe measured 4.0 × 3.8 cm with solid cystic nodules and calcifications, without cervical lymphadenopathy or vocal cord immobility.

Thyroid incidentalomas are found in 1 to 4% of PET/CT scans. Bae et al report a malignancy risk of 30.9% for focal FDG uptake and 6.4% for diffuse uptake. The mean SUVmax for benign nodules is 3.35 ± 1.69, whereas malignant nodules exhibit higher SUVmax (6.64 ± 4.12). With an SUVmax of 3.4, further evaluation via US and fine-needle aspiration was recommended. 12 Chun et al found thyroid incidentalomas with FDG uptake have a malignancy risk of 27.8 to 74%, with malignant nodules showing a median SUV of 4.7 (interquartile range [IQR] 3.4–6.0) and benign nodules of 2.8 (IQR 2.6–4.0). 13 Stangierski et al report malignancy risk at 16.7% for SUVmax < 3.0, 43.8% for SUVmax 3 to 6, and 54.6% for SUVmax > 6. 14 Bolf et al found that women with thyroid cancer have a higher risk of developing breast cancer. 15 Supporting this, a Mendelian randomization study by Tan et al identified a causal relationship between ER+ breast cancer and an increased risk of thyroid cancer (odds ratio [OR] = 1.135, 95% CI: 1.006–1.279, *p* = 0.038), while no significant association was found with TNBC. 16

Several studies suggest a bidirectional association between breast and thyroid cancers. Meta-analyses show an increased risk of thyroid cancer following breast cancer (OR 1.55) and vice versa (OR 1.32). 1 Shared genetic mutations (PTEN, BRCA1/2) and hormonal influences (estrogen receptor expression) contribute to this link. 15 17 Cancer treatments, including radiation therapy and radioactive iodine-131, may further impact risk.

## Conclusion

This case underscores the diagnostic complexities of malignancies with discordant imaging findings. While mammographic and ultrasonographic features strongly suggested breast malignancy, the low FDG uptake on PET/CT emphasized the limitations of metabolic imaging in certain breast cancer subtypes. Similarly, the incidental thyroid lesion with an SUVmax of 3.4 necessitated further evaluation to determine its malignant potential. A comprehensive approach, integrating clinical assessment, imaging modalities, and histopathological confirmation, remains crucial in guiding appropriate management strategies for patients with suspected multiple primary malignancies.
